# Preventing Sexual Transmission of Zika Virus Infection during Pregnancy, Puerto Rico, USA, 2016^1^

**DOI:** 10.3201/eid2511.190915

**Published:** 2019-11

**Authors:** Beatriz Salvesen von Essen, Katie Kortsmit, Lee Warner, Denise V. D’Angelo, Holly B. Shulman, Wanda Hernández Virella, Aspy Taraporewalla, Leslie Harrison, Sascha Ellington, Carrie Shapiro-Mendoza, Wanda Barfield, Ruben A. Smith, Denise J. Jamieson, Shanna Cox, Karen Pazol, Patricia García Díaz, Beatriz Ríos Herrera, Manuel Vargas Bernal, on behalf of the Puerto Rico Department of Health, and the Women’s Health and Fertility Branch, Division of Reproductive Health, National Center for Chronic Disease Prevention and Health Promotion, Centers for Disease Control and Prevention

**Affiliations:** Centers for Disease Control and Prevention, Atlanta, Georgia, USA (B. Salvesen von Essen, K. Kortsmit, L. Warner, D.V. D’Angelo, H.B. Shulman, A. Taraporewalla, L. Harrison, S. Ellington, C. Shapiro-Mendoza, W. Barfield, R.A. Smith, S. Cox, K. Pazol);; Puerto Rico Department of Health, San Juan, Puerto Rico, USA (B. Salvesen von Essen, W. Hernández Virella, P. García Díaz, B. Ríos Herrera, M. Vargas Bernal);; Emory University School of Medicine, Atlanta (D.J. Jamieson)

**Keywords:** pregnancy risk assessment monitoring system, PRAMS, Zika postpartum emergency response study, Zika, Zika virus, viruses, infections, sexual transmission, pregnancy, condom use, prenatal counseling, vector-borne infections, zoonoses, Puerto Rico, United States

## Abstract

We examined condom use throughout pregnancy during the Zika outbreak in Puerto Rico during 2016. Overall, <25% of women reported consistent condom use during pregnancy. However, healthcare provider counseling was associated with a 3-fold increase in consistent use, reinforcing the value of provider counseling in Zika prevention efforts.

Zika virus infection during pregnancy can cause brain abnormalities, microcephaly, and other birth defects in exposed offspring (*1**,**2*). Although transmission of Zika virus primarily occurs through the bite of an infected mosquito, it can also be transmitted by having intercourse with an infected partner (*3**,**4*). In 2016, the Centers for Disease Control and Prevention (CDC) released guidance for prevention of sexual transmission of Zika virus for pregnant women and couples planning to conceive (*3**–**5*). In areas where Zika virus transmission was active, pregnant women and their male partners were advised to consistently and correctly use condoms when having intercourse or to abstain from intercourse during pregnancy to reduce the risk for sexual transmission of Zika virus (*3**–**5*). Corresponding with CDC guidance to healthcare providers (*3**–**5*), the American College of Obstetricians and Gynecologists and the Society for Maternal–Fetal Medicine released interim guidance outlining the need to provide counseling about recommended prevention measures to women and their partners who were at risk for exposure to Zika virus infection (*6*).

## The Study

In 2016, the Puerto Rico Department of Health and CDC partnered to conduct the Pregnancy Risk Assessment Monitoring System–Zika Postpartum Emergency Response Study, a hospital-based survey that collected data from women after delivery and before hospital discharge about their prenatal experiences and behaviors related to detection and prevention of Zika virus infection during pregnancy (*7*). The island-wide study was implemented during August 28–December 3, 2016. Hospitals reporting >100 births during 2015 were eligible to participate. A total of 36 hospitals were eligible and agreed to participate, representing 98% of live births in Puerto Rico.

Women with a recent live birth who were residents of Puerto Rico, had delivered their infant in a participating hospital, and were able to complete the survey in Spanish or English were eligible to participate. To select the study sample, we randomly sampled delivery dates (clusters) within each hospital. All eligible women who delivered their infant on one of the randomly selected delivery dates were invited to participate. Hospital delivery logs were used to identify women for sampling. Sampled women were approached by study staff (24 hours after vaginal deliveries and 36 hours after cesarean deliveries). Overall, of 2,933 women eligible to participate, 2,364 (80.6%) completed surveys.

For women who were sexually active during pregnancy, we assessed the prevalence of condom use during pregnancy, overall and by select maternal characteristics. We constructed 3 separate multivariable logistic regression models to examine factors associated with receiving prenatal provider counseling on condom use for Zika virus infection prevention; any condom use during pregnancy; and consistent condom use during pregnancy. Each model was further adjusted for maternal characteristics, infant birth month (August–September 2016 vs. October–December 2016), and geographic region.

Of 2,229 respondents included in the analysis, most were 20–34 years of age (79.7%), had more than a high school education (68.9%), were unmarried (68.5%), and participated in the Special Supplemental Nutrition Program for Women, Infants, and Children (WIC) during pregnancy (88.3%). Most (80.6%) women reported being sexually active during pregnancy (Figure).

**Figure:**
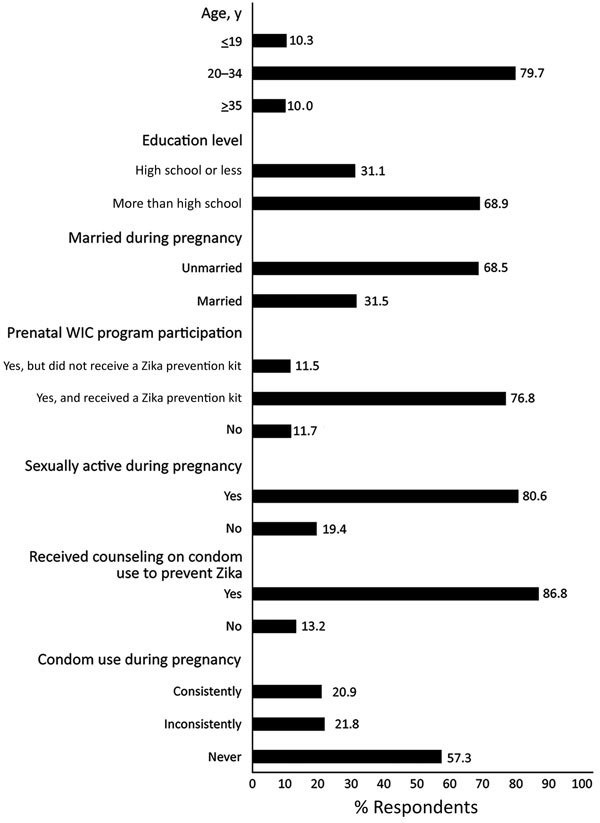
Distribution of maternal characteristics and receipt of counseling on condom use to prevent Zika virus infection, Pregnancy Risk Assessment Monitoring System–Zika Postpartum Emergency Response Study, Puerto Rico, 2016. WIC, Special Supplemental Nutrition Program for Women, Infants, and Children.

Overall, most (86.8%) women reported receiving counseling to use condoms during pregnancy to prevent Zika virus infection. The prevalence of receiving counseling during pregnancy on condom use was significantly higher for mothers <19 years of age (89.7%; adjusted prevalence ratio [aPR] 1.10, 95% CI 1.03–1.18) and those 20–34 years of age (87.3%; aPR 1.07, 95% CI 1.01–1.13) compared with women >35 years of age (81.5%; p<0.01, adjusted linear trend) (Table 1). Receiving counseling during pregnancy on condom use was also higher for women who participated in the WIC program, both those who received a Zika Prevention Kit (containing condoms, repellent, bed nets, and larvicide) from WIC (90.3%; aPR 1.28, 95% CI 1.17–1.40) and those who did not receive a kit (80.2%; aPR 1.14, 95% CI 1.02–1.26) compared with those who did not participate in a WIC program (70.6%).

**Table 1:** Adjusted prevalence estimates and ratios of receipt of provider counseling on condom use during pregnancy to prevent Zika virus infection by maternal characteristics, Pregnancy Risk Assessment Monitoring System–Zika Postpartum Emergency Response Study, Puerto Rico, USA, 2016*

Characteristic	Received counseling on condom use to prevent Zika, n = 2,229†		
	% Patients (95% CI)‡	Crude PR (95% CI)	Adjusted PR (95% CI)§
Age, y			
<19	89.7 (85.6–92.7)	1.17 (1.09–1.26)	1.10 (1.03–1.18)
20–34	87.3 (85.7–88.7)	1.13 (1.06–1.21)	1.07 (1.01–1.13)
>35	81.5 (77.0–85.3)	Referent¶	Referent¶
Education level			
High school or less	86.3 (83.5–88.6)	1.02 (0.99–1.05)	0.99 (0.96–1.03)
More than high school	87.1 (85.4–88.5)	Referent	Referent
Marital status during pregnancy			
Unmarried	86.2 (84.4–87.8)	1.03 (0.99–1.07)	0.98 (0.95–1.01)
Married	88.0 (85.5–90.1)	Referent	Referent
Prenatal WIC program participation			
Yes, did not receive a Zika prevention kit	80.2 (75.4–84.3)	1.13 (1.02–1.24)	1.14 (1.02–1.26)
Yes, received a Zika prevention kit	90.3 (88.9–91.5)	1.27 (1.16–1.38)	1.28 (1.17–1.40)
No	70.6 (64.0–76.5)	Referent	Referent
Sexually active during pregnancy			
Yes	87.0 (85.5–88.3)	Referent	Referent
No	86.3 (83.1–89.0)	1.00 (0.96–1.03)	0.99 (0.96–1.03)

*PR, prevalence ratio; WIC, Special Supplemental Nutrition Program for Women, Infants, and Children. †Unweighted sample size. ‡Adjusted weighted percentage.  §All prevalence and prevalence ratio estimates were adjusted for maternal age and education, marital status, prenatal WIC participation, sexual activity during pregnancy, infant birth month, and region. ¶Adjusted p value (<0.01) for linear trend based on maternal age.

For women who were sexually active during pregnancy, 20.9% used condoms consistently, 21.8% inconsistently, and 57.3% never (Figure). Multivariable analyses (Table 2) showed that the prevalence of any condom use during pregnancy was higher for women with a high school diploma or less (46.8% vs. 41.1%; aPR 1.14, 95% CI 1.02–1.27), those who were WIC program participants and received a Zika Prevention Kit (44.5% vs. 32.3%; aPR 1.38, 95% CI 1.12–1.70), and those who reported prepregnancy condom use (64.7% vs. 40.5%; aPR 1.60, 95% CI 1.42–1.80) compared with their counterparts (Table 2). Receiving healthcare provider counseling during pregnancy regarding the need for condom use was strongly associated with any condom use during pregnancy. Counseled women were >2 times as likely to report any condom use during pregnancy (45.9% vs. 20.4%; aPR 2.25, 95% CI 1.73–2.91) than were noncounseled women (Table 2).

**Table 2:** Adjusted prevalence estimates and ratios of self-reported condom use during pregnancy by maternal characteristics and receipt of provider counseling on condom use during pregnancy, Pregnancy Risk Assessment Monitoring System–Zika Postpartum Emergency Response Study, Puerto Rico, USA, 2016*

Characteristic	Total, n = 1,794†						
	Any condom use				Consistent condom use		
	% Respondents (95% CI)‡	Crude PR (95% CI)	Adjusted PR (95% CI)§		% Respondents (95% CI)‡	Crude PR (95% CI)	Adjusted PR (95% CI)§
Age, y							
<19	51.5 (43.9–59.0)	1.62 (1.29–2.04)	1.22 (0.96–1.54)		24.5 (18.3–31.9)	1.68 (1.12–2.53)	1.13 (0.73–1.75)
20–34	41.8 (39.3–44.3)	1.16 (0.95–1.40)	0.99 (0.82–1.18)		20.4 (18.4–22.5)	1.12 (0.80–1.57)	0.94 (0.67–1.31)
>35	42.4 (35.3–49.8)	Referent¶	Referent		21.7 (15.8–29.1)	Referent#	Referent
Education level							
High school or less	46.8 (42.4–51.4)	1.24 (1.12–1.38)	1.14 (1.02–1.27)		25.2 (21.5–29.2)	1.40 (1.17–1.69)	1.31 (1.07–1.60)
More than high school	41.1 (38.6–43.7)	Referent	Referent		19.2 (17.1–21.5)	Referent	Referent
Marital status during pregnancy							
Unmarried	41.4 (38.8–44.1)	1.03 (0.92–1.15)	0.91 (0.82–1.02)		20.4 (18.4–22.7)	1.05 (0.88–1.26)	0.93 (0.78–1.10)
Married	45.3 (41.4–49.3)	Referent	Referent		22.0 (18.9–25.3)	Referent	Referent
Prenatal WIC program participation							
Yes, did not receive a Zika prevention kit	41.6 (34.9–48.6)	1.32 (1.02–1.71)	1.29 (0.99–1.68)		18.7 (14.1–24.3)	1.08 (0.72–1.61)	0.96 (0.64–1.43)
Yes, received a Zika prevention kit	44.5 (41.9–47.1)	1.52 (1.23–1.87)	1.38 (1.12–1.70)		21.4 (19.3–23.7)	1.36 (1.00–1.86)	1.10 (0.80–1.51)
No	32.3 (26.2–39.0)	Referent	Referent		19.5 (14.4–26.0)	Referent	Referent
Prepregnancy condom use							
Yes	64.7 (57.5–71.2)	1.64 (1.45–1.85)	1.60 (1.42–1.80)		34.7 (28.2–41.9)	1.84 (1.48–2.28)	1.78 (1.44–2.21)
No**	40.5 (38.2–42.8)	Referent	Referent		19.5 (17.7–21.4)	Referent	Referent
Received counseling on condom use to prevent Zika							
Yes	45.9 (43.4–48.4)	2.57 (1.99–3.32)	2.25 (1.73–2.91)		22.8 (20.9–25.0)	3.28 (2.12–5.09)	3.07 (1.97–4.79)
No	20.4 (15.7–26.0)	Referent	Referent		7.4 (4.8–11.4)	Referent	Referent

*PR, prevalence ratio; WIC, Special Supplemental Nutrition Program for Women, Infants, and Children. †Unweighted sample size. ‡Adjusted weighted percentage. §All prevalence and prevalence ratio estimates were adjusted for maternal age, education, marital status, prenatal WIC participation, receipt of provider counseling, infant birth month, and region. ¶Adjusted p value (<0.01) for linear trend based on maternal age. #Adjusted p value (<0.05) for linear trend based on maternal age. **Included women who reported they were not doing anything to prevent pregnancy and women who were using a contraceptive method other than condoms to prevent pregnancy.

Similar to the prevalence of any condom use, the prevalence of consistent condom use during pregnancy was higher for less educated women (25.2% vs. 19.2%; aPR 1.31, 95% CI 1.07–1.60) and those who reported prepregnancy condom use (34.7% vs. 19.5%; aPR 1.78, 95% CI 1.44–2.21). Women who were counseled on condom use were 3 times as likely to report consistent condom use during pregnancy than were noncounseled women (22.8% vs. 7.4%; aPR 3.07, 95% CI 1.97–4.79) (Table 2).

## Conclusions

Although <25% of women reported consistently using condoms during pregnancy, counseling by prenatal care providers was associated with marked increases in any condom use and consistent condom use. Healthcare providers, including doctors, nurses, and other providers in various settings (e.g., prenatal care visits, WIC program visits), can play an important role in prevention of sexual transmission of Zika virus infection by counseling pregnant patients on the importance of consistent and correct condom use. These findings can be used to target and further refine Zika virus prevention messaging and interventions and can apply more broadly to the prevention of other sexually transmitted infections during pregnancy, such as syphilis and genital herpes that, if left untreated, can increase the risk for adverse maternal and infant outcomes (*8**,**9*).

This study also shows how more traditional surveillance systems focused on maternal and child health can successfully be adapted to rapidly collect information from pregnant women during public health emergencies. Interviewing women after delivery and before hospital discharge, although labor- and cost-intensive, can be implemented rapidly with high response rates during urgent situations, such as the Zika outbreak. This type of design might be appropriate for other public health emergencies that affect the health of pregnant women and newborns when the emergency is geographically limited.

## References

[1] Rasmussen SA, Jamieson DJ, Honein MA, Petersen LR. Zika virus and birth defects: reviewing the evidence for causality. N Engl J Med. 2016;374:1981–7. 10.1056/NEJMsr1604338 27074377

[2] Centers for Disease Control and Prevention. Congenital Zika Syndrome and other birth defects; 2018 [cited 2019 Jan 2]. https://www.cdc.gov/pregnancy/zika/testing-follow-up/zika-syndrome-birth-defects.html

[3] Petersen EE, Meaney-Delman D, Neblett-Fanfair R, Havers F, Oduyebo T, Hills SL, et al. Update: interim guidance for preconception counseling and prevention of sexual transmission of Zika virus for persons with possible Zika virus exposure—United States, September 2016. MMWR Morb Mortal Wkly Rep. 2016;65:1077–81. 10.15585/mmwr.mm6539e1 27711033

[4] Polen KD, Gilboa SM, Hills S, Oduyebo T, Kohl KS, Brooks JT, et al. Update: interim guidance for preconception counseling and prevention of sexual transmission of Zika virus for men with possible Zika virus exposure—United States, August 2018. MMWR Morb Mortal Wkly Rep. 2018;67:868–71. 10.15585/mmwr.mm6731e2 30091965 PMC6089331

[5] Oster AM, Russell K, Stryker JE, Friedman A, Kachur RE, Petersen EE, et al. Update: interim guidance for prevention of sexual transmission of Zika virus—United States, 2016. MMWR Morb Mortal Wkly Rep. 2016;65:323–5. 10.15585/mmwr.mm6512e3 27032078

[6] The American College of Obstetricians and Gynecologists Women’s Health Care Physicians, Society for Maternal-Fetal Medicine. Practice advisory interim guidance for care of obstetric patients during a Zika virus outbreak; 2017 [cited 2019 Aug 23]. https://www.acog.org/Clinical-Guidance-and-Publications/Committee-Opinions/Immunization-Infectious-Disease-and-Public-Health-Preparedness-Expert-Work-Group/Management-of-Patients-in-the-Context-of-Zika-Virus

[7] Puerto Rico Department of Health. Puerto Rico Pregnancy Risk Assessment Monitoring System–Zika Postpartum Emergency Response, PRAMS-ZPER 2.0 Protocol; 2017 [cited 2019 Aug 23]. https://www.cdc.gov/prams/special-projects/zika/docs/pdf/english/PRAMS_ZPER-2.0_Protocol_FINAL_508tagged.pdf

[8] Centers for Disease Control and Prevention. Sexually transmitted diseases treatment guidelines; 2015 [cited 2019 Mar 18]. https://www.cdc.gov/std/tg2015/default.htm 10.1093/cid/civ771 26602614

[9] Centers for Disease Control and Prevention. Sexually transmitted disease surveillance; 2016 [cited 2019 Jan 2]. https://www.cdc.gov/std/stats16/CDC_2016_STDS_Report-for508WebSep21_2017_1644.pdf

